# Engrafted Human Induced Pluripotent Stem Cell-Derived Anterior Specified Neural Progenitors Protect the Rat Crushed Optic Nerve

**DOI:** 10.1371/journal.pone.0071855

**Published:** 2013-08-19

**Authors:** Leila Satarian, Mohammad Javan, Sahar Kiani, Maryam Hajikaram, Javad Mirnajafi-Zadeh, Hossein Baharvand

**Affiliations:** 1 Department of Physiology, Faculty of Medical Sciences, Tarbiat Modares University, Tehran, Iran; 2 Department of Stem Cells and Developmental Biology at Cell Science Research Center, Royan Institute for Stem Cell Biology and Technology, ACECR, Tehran, Iran; 3 Department of Developmental Biology, University of Science and Culture, ACECR, Tehran, Iran; University of Iowa Carver College of Medicine, United States of America

## Abstract

**Background:**

Degeneration of retinal ganglion cells (RGCs) is a common occurrence in several eye diseases. This study examined the functional improvement and protection of host RGCs in addition to the survival, integration and neuronal differentiation capabilities of anterior specified neural progenitors (NPs) following intravitreal transplantation.

**Methodology/Principal Findings:**

NPs were produced under defined conditions from human induced pluripotent stem cells (hiPSCs) and transplanted into rats whose optic nerves have been crushed (ONC). hiPSCs were induced to differentiate into anterior specified NPs by the use of Noggin and retinoic acid. The hiPSC-NPs were labeled by green fluorescent protein or a fluorescent tracer 1,1′ -dioctadecyl-3,3,3′,3′-tetramethylindocarbocyanine perchlorate (DiI) and injected two days after induction of ONC in hooded rats. Functional analysis according to visual evoked potential recordings showed significant amplitude recovery in animals transplanted with hiPSC-NPs. Retrograde labeling by an intra-collicular DiI injection showed significantly higher numbers of RGCs and spared axons in ONC rats treated with hiPSC-NPs or their conditioned medium (CM). The analysis of CM of hiPSC-NPs showed the secretion of ciliary neurotrophic factor, basic fibroblast growth factor, and insulin-like growth factor. Optic nerve of cell transplanted groups also had increased GAP43 immunoreactivity and myelin staining by FluoroMyelin™ which imply for protection of axons and myelin. At 60 days post-transplantation hiPSC-NPs were integrated into the ganglion cell layer of the retina and expressed neuronal markers.

**Conclusions/Significance:**

The transplantation of anterior specified NPs may improve optic nerve injury through neuroprotection and differentiation into neuronal lineages. These NPs possibly provide a promising new therapeutic approach for traumatic optic nerve injuries and loss of RGCs caused by other diseases.

## Introduction

The loss of retinal ganglion cells (RGCs) occurs in various eye diseases and injuries, such as glaucoma, ischemia-reperfusion, and traumatic optic nerve crush (ONC). Optic nerve neuropathies that eventually result in irreversible loss of RGCs are commonly observed in young people, leading to a higher socio-economic impact worldwide.

The most widely accepted contemporary treatments for optic nerve neuropathy include pharmacological intervention for reducing or preventing neural damage, reducing or removing the most important risk factors for disease onset and progression, and surgical approaches to decompress the optic nerve [1], [2]. However, despite the availability of treatment progressive visual loss still occurs in a high proportion of patients.

Recent reports on cell transplantation have resulted in new insights into novel possibilities of using stem cells or their derivatives as graft-based therapies to restore optic nerve neuropathies. After the first retinal transplantation in mammals was performed in 1946 by Tansley [3], various in vivo, ex vivo and in vitro studies have been undertaken using different cell sources with the intent to determine the possibility of suitable neuroprotection [4], [5] or to locate an alternative RGC source [6]. However, an efficient, reliable cell source has not been reported, up to now.

The generation of pluripotent human embryonic stem cells (hESCs) in 1998 [7] and human induced pluripotent stem cells (hiPSCs) in 2007 [8] have raised the hopes for curing diseases that have poor prognoses. These pluripotent cells can be a new, promising source of neural progenitors (NPs)/neural stem cells [9], [10] to the regeneration of a nervous system damaged from diseases [11], [12], [13]. Previously, the iPS cells were differentiated to different types of retinal cells under suitable culture conditions [14], [15], [16], [17], [18], [19]. NPs have extensive capacity for proliferation, self-renewal, and differentiation into glial and neuronal lineages.

The generation of RGC-like cells from the pluripotent stem cell-derived NPs in vitro as well as therapeutic use of them in animal retinal models was reported [20], [21], [22], [23]. Therefore, hiPSC-NPs currently offer a more promising strategy for the autologous replacement and restoration of RGC function following irreversible damage. However, there is no report regarding the differentiation of neural cells from hiPSCs and their transplantation into an animal model of optic nerve neuropathy.

On the other hand, successful transplantation mandates in depth knowledge of the genes and factors that control precursor cell development toward a fully functional, differentiated neural cell within a specific CNS region [24]. NPs expressing key genes with the capability to produce a specific cell type or direct desired different pathways are possibly beneficial for transplant therapies that require cellular replacement [25]. Research has demonstrated that retinal neurons and RGCs are mainly comprised of anteriorized NPs that express PAX6 and OTX2 [26], [27]. Therefore, it seems prior to grafting, the establishment of regionally specified NPs that can produce authentic, functionally specific neurons is necessary.

In the present study, we induced hiPSCs to anterior specified NPs and subsequently transplanted them into the vitreous of rats’ eyes that had ONC. We studied the functional integrity of the optic apparatus by using visual evoked potential (VEP) recordings until day 60 post-transplantation. Additionally we researched the possible protective effect of this transplantation on the optic nerve by examining retrograde tracings, optic nerve histopathology, and retinal whole mounts. Finally, we sought to determine if these transplanted cells had the capability to survive, migrate and integrate into the host retina in addition to conducting a study of their fate.

## Methods

### Ethics Statement

All research and animal care procedures were performed according to International Guidelines on the Use of Laboratory Animals, and the Association for Research in Vision and Ophthalmology (ARVO) statements for use animals in ophthalmic and vision research and approved by Tarbiat Modares University Committee for Ethics in Animal Research.

### Culture of hiPSCs and Neural Differentiation

The hiPSC line (Royan hiPSC8, passage 22) [28] was used in these experiments. The colonies were expanded and passaged under feeder-free culture conditions in a pluripotent stem cell medium as previously described [29]. We induced hiPSCs to NPs by a modification of our previously published procedure [9]. Five days after passaging the cells, neural differentiation was begun and performed over a three-week period. We used a basal medium that included DMEM/F12 (Gibco-Invitrogen, 21331-020) supplemented by 5% knockout serum replacement (KOSR), 1% nonessential amino acids (Gibco-Invitrogen, 11140), 2 mM L-glutamine (Gibco-Invitrogen, 25030-024), 2% N2 supplement (Gibco-Invitrogen, 17502-048), 0.1 mM β-mercaptoethanol, and 100 ng/ml bFGF (Royan Institute). During the first week of neural induction, Noggin (100 ng/ml, R&D, 1967-NG) was added to the medium for neural ectoderm induction. In the second week all-trans retinoic acid (RA; Sigma-Aldrich, R2625) at a concentration of 2 µM was added and we increased the concentration of Noggin to 250 ng/ml to induce rosette formation. During the final week Noggin was eliminated from the culture and we continued RA at the same concentration. At this stage, the neural tubes were manually separated and replated on 15 mg/ml poly-L-ornithine (Sigma-Aldrich, P4707) and 1 mg/ml laminin (Sigma-Aldrich, L2020)-coated tissue plates. Expansion medium consisted of basal medium supplemented with bFGF, human epidermal growth factor (EGF, 20 ng/ml, Sigma-Aldrich, E9644) and ascorbic acid (0.2 mM, Sigma-Aldrich, A8960). Medium was replaced on alternate days. NPs cells were grown until approximately 80% confluency and then passaged as necessary.

NPs identity was verified at passages 6 to 9. Spontaneous differentiation was performed in a differentiation medium in the absence of growth factors, but included neurobasal medium and DMEM/F12 at a 1∶1 ratio, 1% B27 supplement (Gibco-Invitrogen, 17504-044), 5% KOSR and 1% N2 supplement. Half of the medium was renewed every 3 days, for a 14-day period.

### RNA Isolation and Real-time Reverse Transcription–polymerase Chain Reaction for Gene Expression Analysis

Total cellular RNA was extracted by TRIzol® reagent (Gibco-Invitrogen, 15596-018) from NPs cells and undifferentiated hiPSCs. After treatment with RNase-free DNase I (Fermentase, END521), 1 µg RNA was reverse transcribed using the RevertAidTM H Minus First Strand cDNA Synthesis Kit (Fermentase K1632). We designed specific human primers (Table S1) and performed real-time PCR analysis with ABI (Applied Biosystems) equipment. For each assay, we run each sample in triplicate. GAPDH was used as the endogenous control. The comparative Ct method (2^−ΔΔCt^) was used to calculate the fold of change in genes expression in hiPSC-NPs compared to their expression in hiPSCs.

### Immunocytoﬂuorescence Staining

NPs were fixed by 4% paraformaldehyde (Sigma-Aldrich, P6148) for 20 min, washed with phosphate buffered saline (PBS), blocked in 5% normal goat serum (Gibco-Invitrogen, 10000 c) and 0.2% Triton™ X-100 (Sigma-Aldrich, T8532) in 0.1 M PBS for 30 min, incubated with primary antibodies (Table S2) that were diluted in blocking solution, overnight at 4°C. After washing, the preparations were incubated with appropriate fluorescent-labeled secondary antibodies (Table S2) for 1 h at room temperature. The first antibodies were omitted in control experiments. Nuclei were counterstained with 4′,6-diamidino-2-phenylindole (DAPI; Sigma-Aldrich, D-8417) and photographed using an Olympus IX71 microscope with a DP72 digital camera and analyzed by LS Software Starter-version 3.2. Immunofluorescence staining was performed for at least three individual cell induction or differentiation experiments. For each experiment we photographed at least seven separate fields and analyzed them for each marker. The percentage of positive cells was determined as the ratio of positive cells to the total number of counted cells that were stained by DAPI.

### Flow Cytometry Analysis

NPs were dissociated in 0.05% trypsin/EDTA (Sigma-Aldrich, 25300). After assaying for cell viability with trypan blue, cells were fixed in an ethanol/acetone solution (ratio: 3∶7) for 10 min at 4°C. We used 1×10^6^ cells for each experiment. Cells were permeabilized at room temperature with 0.1% Triton™X-100 and 10% normal goat serum was used for blocking nonspecific antibody binding. Afterwards, cell suspension was incubated for 1 h with primary antibodies at 4°C, washed three times with PBS, and incubated with the appropriate secondary antibodies (Table S2) for 45 min at 4°C. The percent of positive cells for each marker was determined by a BD-FACS Caliber flow cytometer and data analyzed with WinMDI software.

### Animals and Induction of Optic Nerve Crush

Adult (6 to 8 weeks) hooded male rats (Razi Institute, Karaj, Iran) were kept under a 12 h dark/light cycle with ad libitum access to food and water. Animals were anesthetized with ketamine (50 mg/kg) and xylazine (5 mg/kg), and the optic nerve approached from the orbit by a lateral canthotomy under the guidance of an operating microscope (OMS300, Topcon, California, US). The left optic nerve was crushed at 2.5 mm behind the eye for 30 seconds without inflicting damage to the retinal blood supply. We used a self-closing Castroviejo cross-action forceps (model 35-513-10, Martin Instruments, Tuttingen, Germany) that was modified and calibrated as described by Sautter and Sabel [30]. Antibiotic eye ointment (tetracycline, SinaDarou, Iran) was topically applied to prevent infection.

Intraocular (i.o.) injections of live or dead cells and PBS were made via approaching the dorsal limbus and performed with a 30-gauge needle connected to a 5 µl Hamilton syringe. Animals received a cocktail of three immune suppressant drugs that included ciclosporin (Novartis Pharma AG, Basle, Switzerland), azathioprine (MehrDarou, Tehran, Iran) and prednisolone (Iran Hormone, Tehran, Iran) in their drinking water that was flavored with blackcurrant juice [6]. The cocktail was administrated from two days prior to cell transplantation. Serum levels of ciclosporin were monitored in a random sample of animals and the average concentration was noted to be 1901±159 ng/ml (mean ± SEM; n = 4).

### Visual Evoked Potential Recording

To assess the functional integrity of the rat optic apparatus, we recorded visual evoked potential (VEP) prior to the crush, and at 1, 2, 4 and 8 weeks post-transplantation. VEP recordings were performed as previously described [31], [32]. Briefly, animals were anesthetized and then fixed into a stereotaxic apparatus (Narishige, Tokyo, Japan). A monopolar electrode was implanted on the surface of the occipital cortex (AP: −7.0 and L: +3.0 reffered to Bregma, [33] as the recording electrode and the reference electrode was implanted at anterior part of the skull. The electrodes were connected to a miniature receptacle and fixed with dental cement. For the VEP recording, rats were re-anesthetized and immobilized in a dark box and allowed to adapt to the darkness for 15 min. Flash light stimulation was delivered by a general stimulator (ScienceBeam Co., Tehran, Iran), for 300 times at a frequency of 0.5 Hz. Responses were amplified using a biophysical amplifier (ISO-80, UK) and displayed on a computer monitor. Afterwards, amplified waveforms were averaged and analyzed offline. For each VEP record, we measured the amplitude of the N1P2 wave.

### Cell Labeling and Transplantation

hiPSC-NPs were labeled with either green fluorescence protein (GFP) or a red fluorescence cell tracer, chloromethyl benzamido derivatives of 1, 1-dioctadecyl-3, 3, 3′, 3′ tetramethylindocarbocyanine perchlorate (CM-DiI). For CM-DiI labeling data, please see the Results S1 and Figures S6 and S7.

For GFP labeling, hiPSCs were transfected with linearized pCAG-eGFP-IRES-Puro plasmid by fugene 6 transfection reagent as described previously [34] and selected by puromycin treatment. Colonies retaining constitutive and ubiquitous green fluorescent protein (GFP) expression were picked up mechanically by pipette and expanded individually. The selected subclone with constitutive and ubiquitous GFP expression was differentiated to NPs and characterized as described above.

The inner limiting membrane (ILM) of the retina is composed of mature astrocytes and acts as a barrier against transplanted cells to prevent their efficient integration into the retina. To disturb this barrier, we used α-aminoadipic acid (AAA; Sigma-Aldrich, A0637) as an astrotoxin that has a temporary effect [35]. AAA was suspended in PBS (100 µg/µl) and pH was adjusted to 7.4. For all animals, we injected 5 µl of the AAA solution intravitreally, which was performed simultaneously with induction of the crush. Two days after crushing the optic nerve, 125000 cells were transplanted intravitreally via a 30-gauge needle connected to a 5 µl Hamilton syringe under direct observation by a binocular operating microscope and a glass coverslip coupled to the corneal which was covered by carbomer gel (Liposic, Laboratoire Chauvin, France). The retinal fundus was examined for damage or sign of vascular distress. Any rat that had damage or vascular distress was removed from the study.

### Conditioned Medium (CM) Preparation

hiPSC-NPs were cultured for 48 h in expansion medium after which the CM was collected and centrifuged to obtain a cell-free solution for the in vivo study. Randomly selected rats received intravenous injections of CM (750 µl/rat) every other day from day 2 to 10 post-crush.

### Retrograde Tracing

To evaluate the protective effect of transplanted cells or CM on RGCs, we performed retrograde labeling 21 days after the crush. The fluorescent tracer 1,1′-dioctadecyl-3,3,3′,3′-tetramethylindocarbocyanine perchlorate (DiI; DiIC_18_(3), Molecular Probes, D282, UK) was prepared as a 2% solution in DMSO and injected into the superior colliculi (SCs) in the anesthetized rats. For the injections, rats were placed in a stereotaxic apparatus and we injected 2 µl of DiI into the superficial layers of the SC (AP: 6.0, L: ±1.5, DV: 4.0 mm) via a 30-gauge needle connected to a 5 µl Hamilton syringe. After 5–7 days, the eyes were removed and whole mount retinas prepared. In a random manner, 12 non-overlapping areas (0.03 mm^2^) of each retina, radially distributed at 1–2 mm from the optic nerve head, were selected and photographed at 40×magnification. All DiI-labeled cells with RGC-like morphology were counted and the density of surviving RGCs was determined. Normal controls rats (n = 5) were also labeled and studied to obtain baseline values for RGC density.

### Growth Factor Secretion by hiPSC-NPs

To evaluate growth factor secretion by hiPSC-NPs, we quantified the CM of NPs for protein levels of ciliary neurotrophic factor (CNTF), bFGF and insulin-like growth factor 1 (IGF1) using specific ELISA kits (R&D Systems; DNT00 for CNTF, DFB50 for bFGF, DG100 for IGF1) according to the manufacturer’s instructions. Plated cells were dissociated and counted using a hemocytometer. The results were expressed as picograms or nanograms of growth factor produced per 48 h per 10^6^ cells. The CM of hiPSCs was used as control.

### Tissue Processing and Staining

On different days following ONC, the animals were sacrificed under anesthesia and their eyes and optic nerves harvested. The anterior segment that included the lens was removed. The posterior eyecup and optic nerves were fixed by immersion in 4% paraformaldehyde for 2 h at room temperature, cryoprotected in 30% sucrose/PBS for 24 h at 4°C, and then embedded in optimal cutting temperature compound (OCT/Leica Microsystems). The specimens were cryosectioned into 8-µm sections and mounted on superfrost plus slides (Thermo Scientific, 4951, Germany).

The immunohistofluorescent study was carried out by blocking the sections in PBS that contained 5% normal goat serum and permeabilized with 0.3% Triton X-100 for 1 h at room temperature. Then, sections were incubated with primary antibodies diluted in blocking solution, overnight at 4°C. After three successive washes with PBS/Tween, the slides were incubated with proper secondary antibodies for 1 h at room temperature (Table S2). Nuclei were counterstained with DAPI (Sigma-Aldrich, D-8417).

For FluoroMyelin™ staining the rehydrated cryosections of optic nerves were incubated in staining solution for 20 min according to the manufacturer’s protocol (Molecular Probes, F34652). Images were obtained with an Olympus BX51 microscope that had a DP72 digital camera and captured by Analysis LS Starter (version 3.2).

To assess the axonal loss in the crushed nerve, optic nerves were harvested and immersed in 4% paraformaldehyde/5% glutaraldehyde (TAAB, G002, England) prepared in PBS for seven days at 4°C, post-fixed in 1% osmium tetroxide (TAAB, O002) for 3 h, dehydrated and embedded in araldite resin (Araldite CY212, TAAB, E009) for semi-thin sectioning. Semi-thin transverse sections were cut from the nerve, at a distance of 2–3 mm distal to the globe, dried on slides, and stained with toluidine blue.

### Statistical Analysis

For qRT-PCR studies, randomization tests that used REST 2009 V2.0.13 software were performed to determine statistically significant changes in the expression of genes in neural progenitor cells compared to the hiPSC. All quantitative data and the difference between the amplitude recoveries of the N1P2 waves in different groups were evaluated by a mixed model of ANOVA with a repeated factor of time and a non-repeated factor of groups and LSD post-hoc. The number of surviving RGCs was normalized to the mean of data obtained from intact animals and compared using unpaired two-tailed student’s *t*-test. Data were expressed as mean ± SEM. P values <0.05 were considered statistically significant.

## Results

### Generation of Anterior NPs from hiPSCs

**Figure 1:**
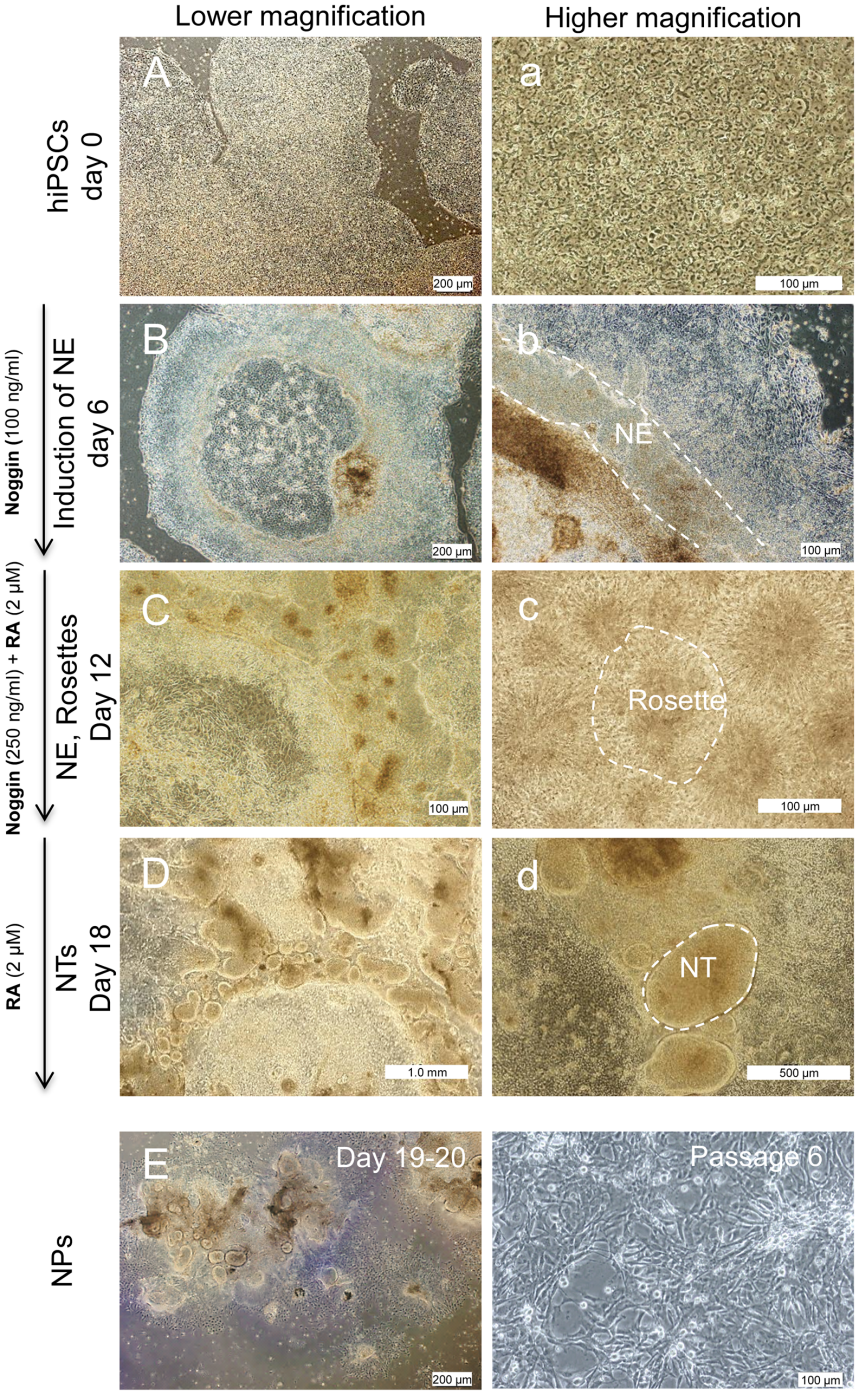
Differentiation protocol and changes in cell morphology during neural progenitor induction. Light microscopy images show the differentiation process of hiPSC line 8 (A,a) into neural ectoderm (NE; B,b), rosette formation (C,c) and neural tube-like structure (NT; D,d) over three weeks. To expand neural progenitors (NPs), the NT structures were mechanically isolated using an inverted phase contrast microscope at day 18 and replated on poly-l-ornithine/laminin-coated plates (E), then passaged every 4–6 days. (F) The morphology of cells at passage 6.

To produce self-renewing anterior NPs, hiPSC8 line was cultured and differentiated under feeder- and serum-free conditions (Figs. 1A and 1a). hiPSCs were induced into neural differentiation in defined adherent culture in the presence of Noggin (100 ng/ml) for six days (Figs. 1B and 1b) and followed in same medium with increasing concentrations of Noggin supplemented with RA, then further cultured without Noggin for an additional six days.

During the initial six-day induction, the cells exhibited the first sign of neural differentiation, the appearance of columnar cells (Figs. 1B and 1b). In the second step, the columnar cells formed rosette structures (Figs. 1C and 1c). In the final step, neural ectodermal cells organized into neural tube-like structures with lumens (Figs. 1D and 1d).

We used a pipette to harvest hiPSC-generated neural tube-like structures under phase contrast microscopy in order to reduce contamination by other cells. The isolated tubes were replated in the presence of bFGF and EGF on poly-l-ornithine/laminin -coated plates (Fig. 1E). After six days, the expanded clusters were dissociated into single cells using a 0.008% trypsin/2 mM EDTA solution and plated in a fresh defined medium in the presence of bFGF/EGF on a poly-l-ornithine/laminin substrate for one week. In the same manner, we passaged the cells at ratios of 1∶2 to 1∶3 every four-six days, then analyzed and transplanted them at passages 6–9. Under these conditions, the cells generated a monolayer that had a homogeneous morphology (Fig. 1F).

### Characterizing hiPSC-NPs

**Figure 2:**
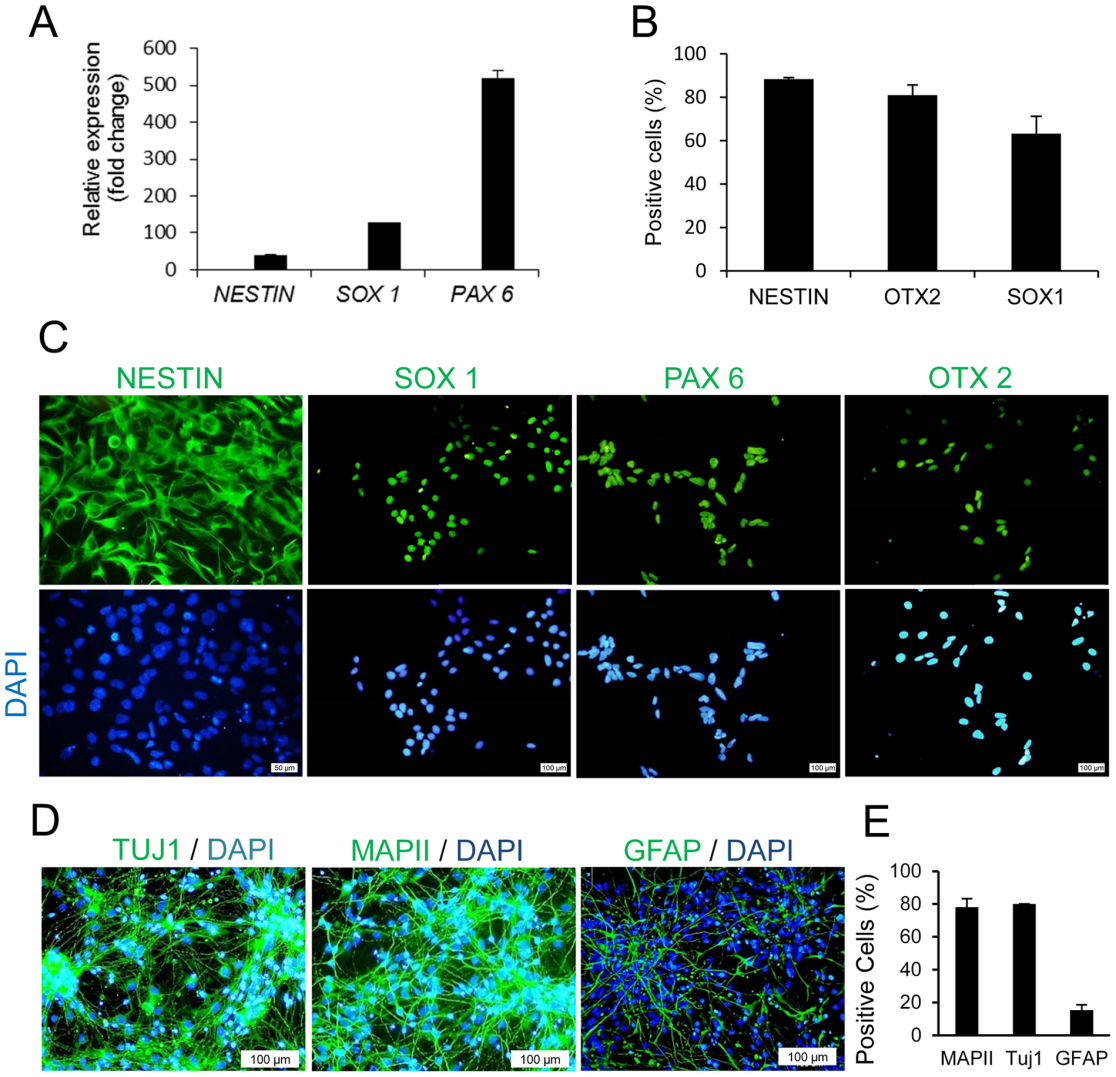
Characterization of hiPSC-NPs in vitro. (A) Real-time PCR shows changes in the expression a set of neural progenitor (NP) gene markers. Data shows increased expression of *NESTIN*, *SOX1,* and *PAX6* relative to hiPSCs as averaged from three independent experiments. (B) The number of positive cells for NP markers was determined by flowcytometric analysis, which revealed a high percentage of NPs that expressed NESTIN, OTX 2, and SOX1. Sample FACS histograms are presented as Fig. S1. (C) Immunofluorescence staining of hiPSC-NPs showed high expression levels of NESTIN, SOX1, PAX6, and OTX2. The blue stain represents nuclear counterstaining with DAPI. (D) Immunofluorescence staining following spontaneous differentiation of hiPSC-NPs. The representative micrographs of MAPII, TUJ1 as neural markers, and GFAP as a glial marker show a high percentage of neural differentiation. (E) Quantitative data for the percent of positive cells following spontaneous differentiation.

To characterize hiPSC-NPs, the expression pattern of a subset of NP marker genes was studied by qRT-PCR (Fig. 2A). Expression levels of *NESTIN* and *SOX1* (NP markers) and *PAX6* (eye field specification marker), which were calculated as fold of change compared to those of hiPSCs, were significantly increased (*p*<0.001).

For further analysis, we performed flow cytometry against the NP markers. As seen in Figure 2B, data analysis revealed that the cell population expressed NESTIN (84±5%), OTX2 (79±4%), and SOX1 (60±10%). Sample histograms are presented in figure S1.

Immunofluorescent analysis demonstrated the expression of neural lineage markers that included NESTIN, SOX1, PAX6 and OTX2 (Fig. 2C). OTX2, a marker of anterior forebrain fate, [36] was expressed in the majority of differentiated cells. Only secondary antibody was used to rule out non-specific staining (Figure S2).

To assess neural differentiation potential of hiPSC-NPs, we withdrew the GFs to enable the cells to undergo spontaneous differentiation. At 14 days following withdrawal of the GFs, we assessed for specific markers of neurons (MAPII and TUJ1) and astrocytes (GFAP). Quantitative analysis demonstrated that more than 78% of differentiated NPs expressed MAPII, 80% were positive for Tuj1, and 15% were GFAP-positive (Figs. 2D and 2E).

To confirm the induction protocol, we repeated this assessment using a different hiPSC line, hiPSC4 [28] and got similar results (data not shown). Therefore, we continued our experiments with hiPSC8-NPs.

### Functional Recovery in Crushed Optic Nerve of Rats Following hiPSC-NPs Transplantation

Because we observed neuronal differentiation of hiPSC-NPs in vitro, we hypothesized that transplantation of the hiPSC-NPs could improve the functional recovery in ONC of rats by neuronal differentiation in vivo or by paracrine effects. Using a dissecting microscope, we injected labeled NPs intravitreally. Figure S3 shows the toluidine blue stained semi-thin sections of intact and ONC on day 14. Staining of the sections obtained from the optic nerve proximal to the crushed site showed a distinct axon degeneration and reduced number of axons. We observed that >75% of axons were lost accompanied by myelin destruction.

**Figure 3:**
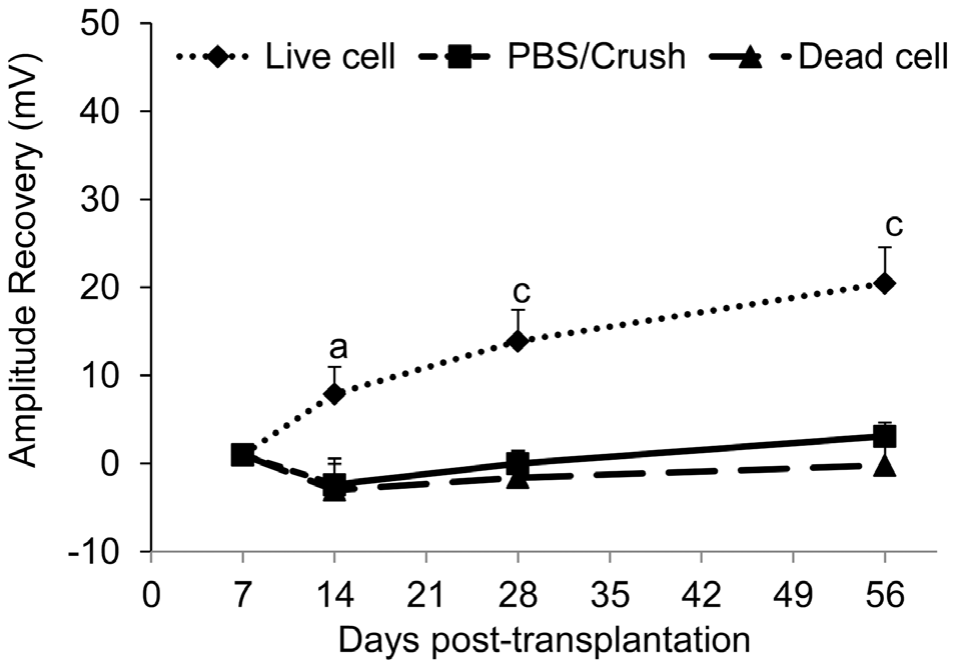
Functional assessment of optic nerve repair and integrity using visual evoked potential recording. Changes in amplitude of the N1P2 wave was evaluated at eight weeks post-injury in vehicle, live cells and dead cells transplanted groups. Changes are calculated as post-transplantation (TX) recovery in the N1P2 amplitude. Significant recovery was observed in the live cell transplanted animals. The average of amplitude was equal to 106.7 mv for the base line and 25.5 mv for day 7. a: *p*<0.05, c: *p*<0.001, data represent mean±SEM, n = 8 per group.

VEP recordings reflect functional integrity of the visual pathway up to the visual cortex. The protective effect of cell therapy and its effect on the repair process of the optic nerve were evaluated using VEP recordings at 1, 2, 4 and 8 weeks post-transplantation. The amplitude of the VEP waves correlate with the number of axons participating in signal transmission. The N1P2 wave was the most stable recorded wave that had large amplitude in the control rats (baseline value = 106.7 mv). Although the amplitude of this wave significantly reduced on day 9 following the crush (equal to 1 week after transplantation) in all groups (average value = 25.5 mv) there was no significant difference between the groups. The amplitude continued to be reduced in subsequent days in vehicle- or dead cell-treated animals. However in the hiPSC-NPs transplanted animals, we observed a significant increased trend in the amplitude of the N1P2 wave. This amplitude significantly increased in hiPSC-NPs treated animals on 2 (*p*<0.05), 4 and 8 (both *p*<0.001) weeks post-infliction of the lesion (Fig. 3).

As a result of the functional recovery of the optic nerve as detected by the VEP recording, we attempted to determine the possible mechanisms of action of the transplanted hiPSC-NPs. We studied the possible protection of RGCs and their axons, optic nerve myelination, and possible differentiation of hiPSC-NPs to RGCs.

### hiPSC-NPs Protect RGCs, Axons, and Myelination

**Figure 4:**
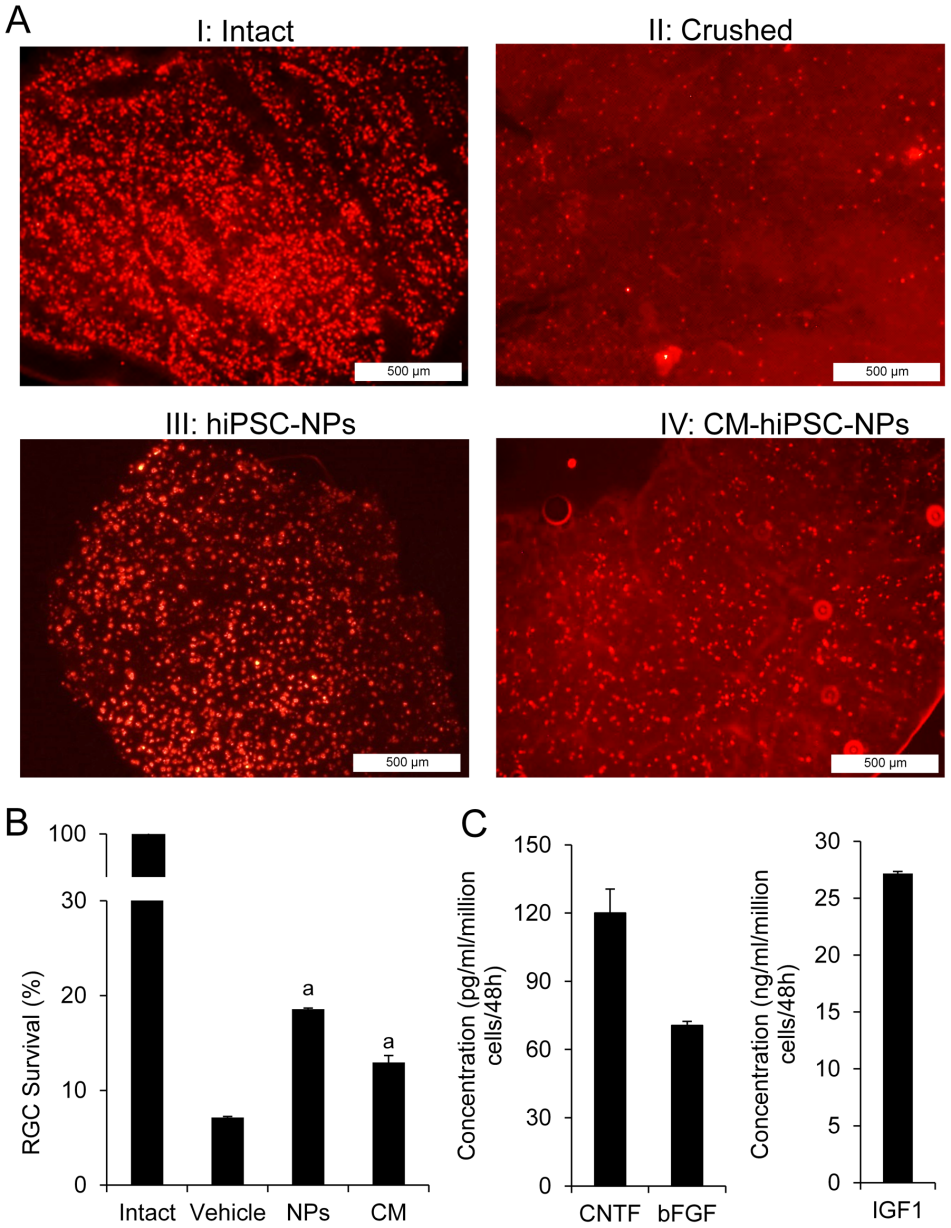
Neuroprotective effects of hiPSC-NPs transplantation and its conditioned medium (CM) on retinal ganglion cells of crushed optic nerves. Retrograde labeling by intracollicular injection of DiI showed labeled retinal ganglion cells (RGCs) in the retina. (A) Representative micrographs of retrograde-labeled RGCs in different experimental groups including (A*I*) animals with intact optic nerve; (A*II*) animals with optic nerve crush (ONC); (A*III*) animals with ONC and transplanted cells; and (A*IV*) animals with ONC and treated with CM. For higher magnification images, please see figure S4. (B) Quantitative analysis of RGC survival as number of cells/mm^2^ in the retina of different groups on day 21 post-ONC, n = 4, a: *p<*0.05 as determined by student *t*-test. Compared to the intact nerve, a few cells were labeled in the eye with vehicle-transplanted nerve following the crush. Both hiPSC-NPs transplantation and CM of cells (CM-hiPSC-NPs) protected RGCs and increased the remaining cell population. (C) CM of hiPSC-NPs evaluated by ELISA for the secretion of trophic factors. CNTF, FGF2 and IGF1 were released into the medium. Data are expressed as mean±SEM, n = 9 independent experiments.

In an attempt to study the neurotrophic effect of hiPSC-NPs on ONC, we evaluated the effects of cell transplantation and the injection of CM from hiPSC-NPs on RGC survival and their projections to the brain. A retrograde tracing approach that used microinjections of DiI into the SCs and its detection within the RGCs layer of retina was used. The number of labeled RGCs within the retina was quantified and compared between groups. In animals with intact visual pathway, labeled cells density was 1930±40 RGCs/mm^2^. Three weeks after crushing the nerve, we observed a huge decrease in the number of labeled RGCs (Fig. 4A, cell density = 138±9 RGCs/mm^2^). Animals that had ONC and received hiPSC-NPs had higher densities of labeled RGCs in their retinas (358±70 RGCs/mm^2^, Fig. 4A). hiPSC-NP CM protected the RGCs and increased the number of labeled cells to 250±28 RGCs/mm^2^ (Fig. 4A). Higher magnification images from labeled RGCs are presented in figure S4. Fig. 4B compares the quantitative data for the density of retrograde-labeled cells in the retinas of animals in different groups. The number of labeled cells in cell transplanted and CM-treated groups significantly increased (both *p*<0.05).

We analyzed the possible secretion of some factors with potential neuroprotective effects in the brain and retina by hiPSC-NPs. CM was collected 48 h post-seeding and evaluated by ELISA for CNTF, FGF2 and IGF1. The concentrations of released factors were: 120.0±10.0 pg/ml (CNTF), 70.5±1.8 pg/ml (bFGF), and 27.1±0.1 ng/ml (IGF1) per 10^6^ cells per 48 h (Fig. 4C).

**Figure 5:**
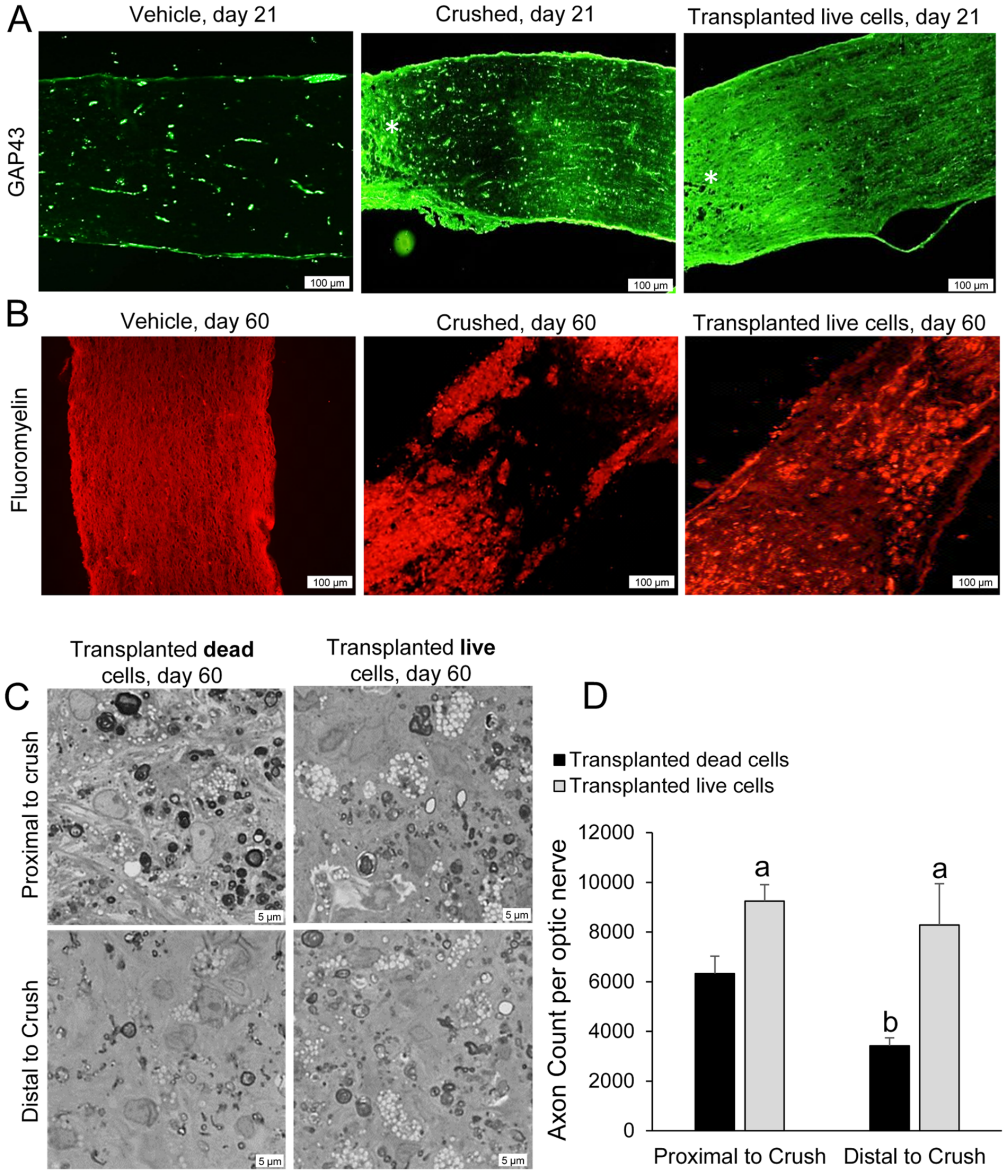
Axon protection by transplanted cells. (A) GAP43 was up-regulated in the vehicle transplanted crushed optic nerve on day 21, whereas the control optic nerve rarely showed GAP43 staining. At the same time point, the cell transplanted group showed higher GAP43 staining at the site of the crush. *: Crush site. (B) The myelination level of the optic nerve was assessed at the site of crush by FluoroMyelin™ staining of the cryosections. While a huge demyelination was observed in the vehicle-transplanted group, cell-transplanted animals showed higher myelin staining at the site of the crush, presumably due to axonal protection and prevention of secondary demyelination. (C) Axon protection was assessed on day 60 post-transplantation using toluidine blue staining of osmium tetroxide-embedded optic nerves in the semi-thin sections. While the crush reduced the number of axons in the both proximal and distal parts of the lesion in animal treated with dead cell as control or live cells, higher number of axons were protected in the optic nerve of animals treated with hiPSC-NPs transplantation. (D) Quantitative analysis of the preserved axons showed higher number of axons in both proximal and distal parts of lesion in hiPSC-NPs treated rats. a: p<0.05 vs. corresponding site in dead cell treated group, b: p<0.05 vs. proximal to crush in dead cell treated animals.

To evaluate the population of regenerating axons following the transplantation of hiPSC-NPs, we assessed GAP43 reactivity in the crushed optic nerves by immunofluorescent staining. GAP43 is a marker of regenerative neurons with an axon outgrowth [37]. Intact optic nerves obtained from animals with intravitreal injections of PBS did not show GAP43 reactivity (Fig. 5A). At day 21, some extent of reactivity to GAP43 antibody was observed at the crush. Animals that received both ONC and transplanted hiPSC-NPs showed higher GAP43 reactivity with extensions into the lesion and toward the distal optic nerve.

Figure 5B shows FluoroMyelin™ staining of the myelin of optic nerves in intact, ONC, and ONC animals treated with transplanted hiPSC-NPs. A higher level of myelination was observed in the crush site of optic nerves obtained from cell-transplanted animals 60 days after cell transplantation.

The axons that remained in the optic nerve in both proximal and distal (500 nm proximal or 500 nm distal) to the crush site were histologically examined by toluidine blue staining of semi-thin sections at day 60 post-lesion. Compared with the number of axons in the intact right optic nerve, a high proportion of axons were lost in the left side crushed optic nerve (Fig. 5C). Cell transplantation increased the number of axons in the crushed optic nerve and the protected axons were mostly visualized as clusters. The axon counts are presented in figure 5D. Higher number of axons was observed in the cell transplanted animals in both proximal and distal parts of crush (p<0.01 and p<0.05, respectively). When the post crush sites were compared, the cell transplanted animals showed higher axons count (p<0.05).

### Integration and Differentiation of Engrafted Cells in the Retina

Figure S5 shows the integration of GFP-labeled NPs inside the retina. The integration of NPs into the retina was examined on flat, whole mounted retina at day 30 post-transplantation. The figure shows a number of the transplanted cells inside the whole mount preparation of the retina.

**Figure 6:**
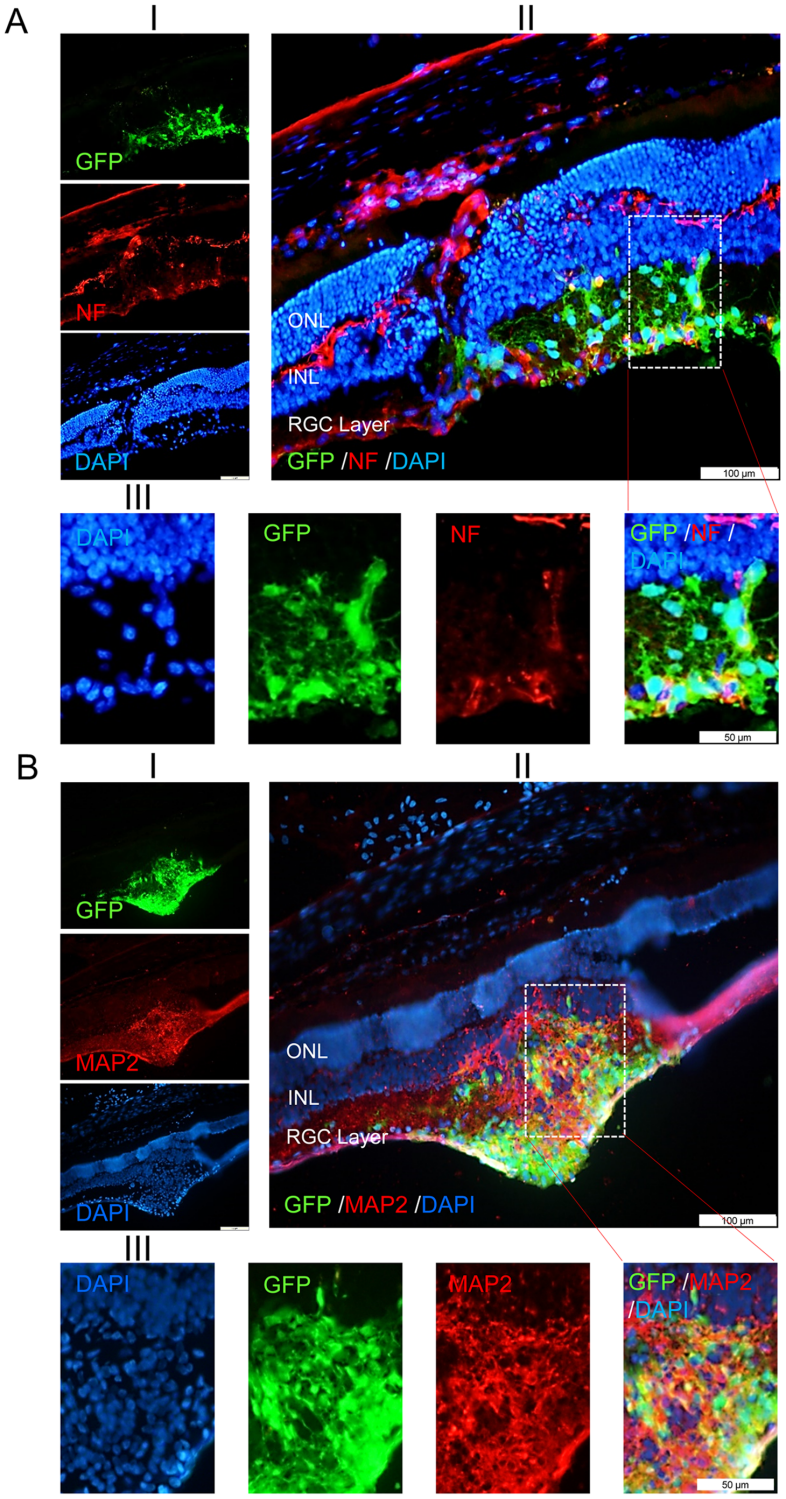
Integration and differentiation of hiPSC-NPs into the retinal ganglion cell layer of rat eyes with optic nerve crush. Integration of GFP-labeled hiPSC-NPs into the retina and their differentiation to the neuronal cells at day 60 post-transplantation. Neuronal differentiation was detected using anti-NF200 (A) and anti-MAPII (B) in the retinal cryosections. Double labeled cells were present inside the RGC layer. In parts A and B, (I) shows the micrographs labeled with GFP, related specific antibody and DAPI; (II) shows the overlay of 3 micrographs mentioned in (I); and (III) shows the higher magnification of rectangle area in part (II).

In another experiment, we further evaluated GFP^+^ transplanted cells, by performing immunohistofluorescent staining against specific markers of RGCs on day 60 post-transplantation (Fig. 6). Figure 6A shows the presence of NF200/GFP^+^ cells inside the ganglion cell layer which implied that transplanted hiPSC-NPs were further differentiated along the neuronal cell lineage. Same observations were made using another neuronal marker, MAPII (Fig. 6B). Thus, these findings showed the survival and the adequate localization and neuronal differentiation of transplanted cells at day 60 post-injection.

**Figure 7:**
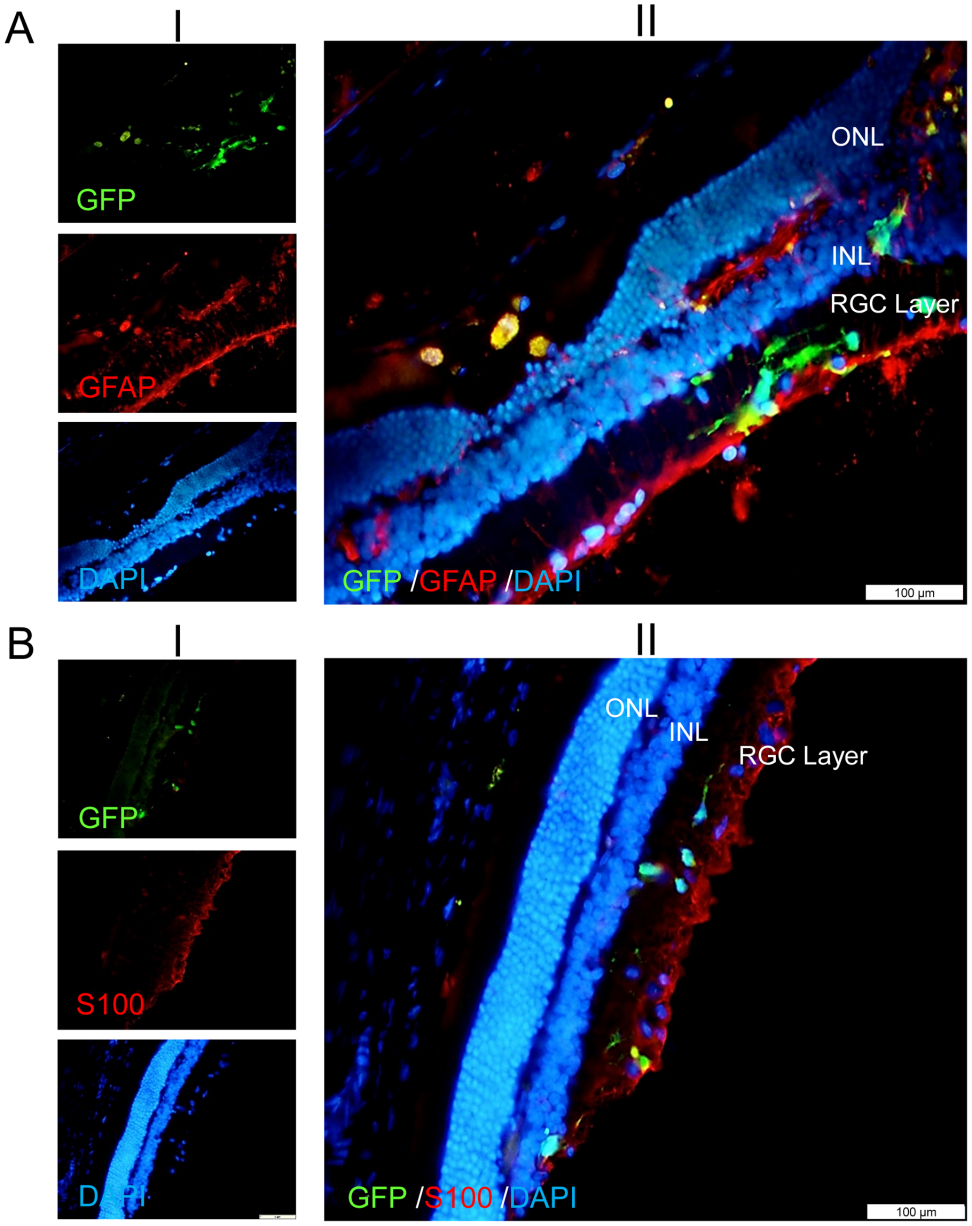
Evaluating the possible glial differentiation of hiPSC-NPs following integration into the retinal ganglion cell layer of rat eyes with optic nerve crush at day 60 post-transplantation. No remarkable staining with GFAP (A) or S100 (B) was observed for GFP-labeled hiPSC-NPs which were integrated into the retinal ganglion cells (RGCs) layer. In parts A and B, (I) shows the micrographs labeled with GFP, related specific antibody and DAPI; (II) shows the overlay of 3 micrographs mentioned in (I).

We also evaluated the possible differentiation of GFP positive transplanted hiPSC-NPs to glial cells. The evaluation was performed using antibodies against GFAP and S100 antigens. As it is mentioned in figure 7, no remarkable glial differentiation was observed using GFAP (Fig. 7A) or S100 (Fig. 7B) staining.

In addition, in a parallel experiment we evaluated the survival, localization and neural differentiation of transplanted cells in vivo using CM-DiI labeling (Results S1). Our results showed the survival and integration of labeled cells into the retina on day 14 post-transplantation (Fig. S6). Further evaluation on day 60 post-transplantation showed their differentiation into MAPII-positive and βIII tubulin-positive cells (Fig. S7).

No evidence of tumor formation was shown in the transplanted groups after eight weeks. Therefore, although teratomas are predominantly derived from hiPSC grafts, purified hiPSC-NPs provide an opportunity for cell-based therapies.

## Discussion

In this study, the anterior-specified NPs derived from hiPSCs were able to protect and/or integrate into the retina with degenerating RGCs. VEP recordings were performed to assess functional analysis of the outcomes. RGC loss is a common hallmark of numerous degenerative eye diseases such as glaucoma, diabetes, Leber’s hereditary optic neuropathy, ischemic optic neuropathy and trauma such as optic nerve compression and crushed optic nerves [for review see [38]].

Several attempts have been undertaken to identify a safe and efficient source of cells that have the capability to replace RGCs. The derivations of hiPSCs provide an attractive opportunity to restore vision after loss or irreversible damage to RGCs by replacing them with an autologous transplantation.

The present study demonstrated that hiPSCs efficiently differentiated into NPs by the BMP- antagonist Noggin and RA. The NPs expressed NESTIN, SOX1, and PAX6 and had the potential to differentiate into neural cells in vitro. Additionally, the differentiated NPs had high expression levels of PAX6 and OTX2 markers for anterior brain and retinal differentiating linage cells [26], [27]. We have previously shown that lower passages of generated NPs from pluripotent stem cells could express transcription factors that were mostly compatible with forebrain and rostral identity [25] and could mimic the default model with rostral or anterior identity in vivo.

Then, hiPSC-NPs were transplanted intravitreally into a rat model of ONC. The ILM of the retina which is constructed by laterally contacting Muller cell end feet and the basement membrane is the main barrier for penetration of intravitreally transplanted cells into the retina. For this reason even the most successful transplantations report that approximately 1% of transplanted cells migrate into the retina [39]. Johnson et al. have demonstrated improvement in retinal engraftment of intravitreally transplanted cells after destroying ILM with L-AAA, an astrotoxin which selectively kills astrocytes, yet minimally impacts surrounding neurons [40], [41]. Maximum defeat of ILM occurs on day 2 post-AAA injection, with an almost a complete recovery observed on day 14 [35]. Therefore, in the current study we applied AAA, two days prior to cell transplantation. To confirm ONC, using toluidine blue staining of osmium tetroxide-embedded optic nerves, we demonstrated a huge reduction in optic nerve axons in the vicinity of the lesion.

VEP recordings provide useful information regarding the functional integrity of the optic apparatus by primarily detecting RGC function and the upstream pathway, thereby determining optic nerve function [42]. While the delay of VEP components is more sensitive to the myelination status of the pathway, the amplitude is more sensitive to the number of functional axons. In the current study we have evaluated changes in amplitude of the N1P2 wave. The greatest decrease in amplitude in the cell-transplanted animals was observed on day 7 post-transplantation, thus we calculated the amount of amplitude recovery in the subsequent recording days for each animal by subtracting its amplitude on day 7. VEP recordings showed that hiPSC-NPs transplantation caused a significant recovery in function of the injured optic nerve on weeks 2, 4 and 8. There was no functional rescue noted with injections of PBS or dead cells, thus refuting any nonspecific effect of cell transplantation and the possible neuroprotective effect that arises from an inflammatory response triggered by i.o. injection of cells.

VEP improvement may be related to the immunomodulatory or trophic effect of transplanted NPs on neural protection [43]. This protection was also observed following CM injection. The difference between cell-transplanted and CM-treated groups may be due to continued secretion of NTFs by NPs and/or a more effective secretion of neurotrophic factors by NPs engrafted into the RGC layer. NPs are known to secrete a variety of growth factors [44]. Previously, the protective effect of human iPS-derived retinal pigment epithelium cells was reported in retinal dystrophic rats [14]. Our results have shown in vitro secretion of a set of neurotrophic factors, IGF1, bFGF and CNTF by hiPSC-NPs. It was reported that intravitreal transplantation of fibroblasts which expressed a combination of neurotrophic factors enhanced neural survival and promoted axonal regeneration [4]. Injections of dead cells were ineffective on VEP recordings which proved that trophic factors were actively secreted by injection of live cells. A variety of neurotrophic factors have been shown to prevent cells from apoptosis and retinal degeneration [45], [46]. In a permissive environment, adult mammalian RGCs that have been protected from death possess the capacity to repair and regenerate axons.

Protection of RGCs and their axons were evaluated using retrograde tracing by injecting CM-DiI into the SCs and evaluating the number of stained RGCs within the whole mount retina after 5–7 days. While the density of labeled RGCs dramatically decreased in untreated animals, both hiPSC-NPs transplantation and injection of CM rescued the RGCs and increased RGCs density in the crushed nerve.

Based on reports, the earliest staining for GAP43 as a marker of regenerating axons has been reported on day 21 post-ONC [37]. While there was no GAP43 immunoreactivity in the intact optic nerve, a low level of immunoreactivity was observed on the proximal side of the injured optic nerve in the vehicle group. Transplantation of hiPSC-NPs or intravenous injection of CM significantly increased GAP43 reactivity and proved the protective effect that has been suggested for transplanted NPs. The higher amounts of GAP43 reactivity seems to be due to higher number of protected axons. Considering the low numbers of integrated neurons within the retina, the increased numbers of GAP43 axons may not be obtained from newly integrated neurons and the stained neurons predominantly represent protected RGCs.

Analysis of transplanted cells indicated that some transplanted hiPSC-NPs were able to survive and migrate across the ILM and incorporate within the degenerating retina. The majority of incorporated cells had differentiated into cells with a neural-like morphology. We also demonstrated that transplanted cells survived at least for 60 days, associated closely with or incorporated into the degenerating retina, and prominently differentiated in vivo into cells with neuronal-like morphologies, largely expressed neuronal markers that included MAPII, NF200 and β III-tubulin. Therefore, the niche of the eye that suffered from the ONC and subsequent RGCs degeneration seemed to promote long-term survival and efficient neural differentiation of hiPSC-NPs.

Neuronal differentiation is more effective when the cells are placed in areas of the CNS that contain reduced numbers of neurons or an ongoing injury [47]. NPs respond to the local signals provided by the niche due to the loss of neurons in neural injuries. In our study we have shown that NPs differentiated into cells which expressed specific markers of neural cell lineage and suggested that the cues in this specific microenvironment supported hiPSC-NPs differentiation into further matured neuronal cells.

Additionally, in another CNS neural depletion model optimal engraftment was achieved when cells were transplanted during the period of cell death. In previous reports, Banin et al. have shown that transplantation of hESC-NPs, either subretinal or intravitreal, into mice led to their integration into the retina where they survived for a long period after grafting, although most displayed photoreceptor markers [48]. Transplantation of a neural stem cell (NSCs)-like clonal line in a rapid RGC-depletion model increased cell incorporation into the RGC layer, however the grafted cells did not differentiate into an RGC phenotype [49]. To date, only a few studies have investigated the potential effect of iPSCs in the replacement of neural cells in retinal degenerative diseases. Parameswaran and colleagues have reported that mouse iPSCs could give rise to both RGCs and photoreceptors in vitro [21]. Recent evidence has shown that mouse iPSCs have the capability to differentiate into RGC-like cells that expressed markers of retinal progenitor cells in vitro. Following transplantation and further differentiation, they expressed mature RGC markers but the transplanted cells did not engraft into the murine retina following intravitreal injection [23].

Our results showed that anterior specified NPs derived from hiPSCs were able to differentiate into neuronal cells both in vitro and in vivo when they were grafted into an ONC animal model. The grafted cells showed significant protective effect on the affected RGCs and improved the functional integrity of the optic apparatus by preserving RGCs and their axons. These grafted cells were able to pass through the ILM of the retina, integrate into the RGC layer and differentiate into neuronal phenotype. The transplanted cells may improve optic nerve injury through stem cell-mediated neuroprotection and differentiation into neurons. Future complementary interventions which allow the integrated neurons to be able to extend their axons within the myelinated optic nerve toward downstream optical structures hold promise for their application in replacing RGCs loss in a variety of eye diseases.

## Supporting Information

Figure S1 **The histograms of hiPSc-NPs and hiPSCs analysis by flow cytometry.** The averaged data are presented as figure 2B.
(TIF)

Figure S2 **Immunofluorescence assessment to rule out non-specific staining by second antibody (2nd Ab-FITC).**
(TIF)

Figure S3 **Semi-thin cross sections of optic nerves.** For verifying the success of the optic nerve crush (ONC), we used toluidine blue staining of osmium tetroxide-embedded optic nerves that were obtained from intact or modeled animals on day 14 post-lesion. The staining protocol is described in the main text. Representative high magnification micrographs of healthy control and crushed optic nerve at two weeks post-lesion. Semi-thin cross sections of crushed optic nerves showed more than 75% axonal loss and extensive demyelination.
(TIF)

Figure S4 **Retrograde-labeled RGCs.** RGCs were retrogradely labeled using DiI injection into the superior colliculi for comparing the number of RGCs with intact axons in different groups. Treatment with both hiPSc-NPs and conditioned medium (CM) were able to increase the protected cells. Low power figures and quantitative data are mentioned in Figure 4 A and B.
(TIF)

Figure S5 **The integration of hiPSC-NPs into the retina of rat eyes with optic nerve crush.** GFP-labeled hiPSC-NPs were traced at day 30 post-transplantation. Whole mount retina preparation of transplanted eye shows the integration of labeled cells into the retina. Transplanted cells showed neural morphology with considerable neurite outgrowths. Data for neural differentiation of the GFP-labeled transplanted cells are presented in Figure 6.
(TIF)

Figure S6 **Fluorescent labeling of hiPSC-NPs and their localization after transplantation into the retina.** (A) The labeled cells in vitro. (B) Large clusters of DiI-labeled hiPSC-NPs that survived within the vitreous after three days. (C) Some transplanted hiPSC-NPs migrated and localized in the proximity of the RGC layer at day 14 post-transplantation. (D) Whole mounted retina visualized 14 days after cell transplantation shows integrated cells. Arrows show transplanted cells and blue shows the nuclear staining using DAPI. L; Lens, V; Vitreous, R; Retina, ONL; Outer nuclear layer, INL; Inner nuclear layer, RGC; Retinal ganglion cell layer; ONH, Optic nerve head.
(TIF)

Figure S7 **The integration and differentiation of hiPSC-NPs at 60 days after transplantation.** Engrafted hiPSC-NPs were labeled by DiI (red fluorescent) and detected using immunohistoflourescence studies against neural markers MAPII and Tuj1 and counterstained with DAPI (blue). DiI^+^/MAPII^+^ cells or DiI^+^/Tuj^+^ (arrows) show that transplanted cells integrated into the RGC layer and underwent neural differentiation (A and B). Immunohistoflourescence evaluation of the retina sections confirmed that transplanted cells localized in the RGC layer and differentiated toward neurons and protruded fine neurite-like processes that elongated directly toward the optic nerve head (B, magnified in inlet). (C) Arrows show that some of the transplanted cells could participate in inner limiting membrane (ILM) repair and expressed the astrocyte/Muller cell marker GFAP. ONL; Outer nuclear layer, INL; Inner nuclear layer, RGC; Retinal ganglion cell layer.
(TIF)

Table S1 **Details of primers used for real time-PCR.**
(PDF)

Table S2 **Details of antibodies and fluorescent markers.**
(PDF)

Results S1 **Determining the fate of grafted cells in the host retina using DiI labeling.**
(PDF)
