# Supplementary figures and images for: Engrafted Human Induced Pluripotent Stem Cell-Derived Anterior Specified Neural Progenitors Protect the Rat Crushed Optic Nerve
Source: PLoS One. 2013 Aug 19;8(8):e71855. doi: 10.1371/journal.pone.0071855 (PMC3747054; doi:10.1371/journal.pone.0071855)

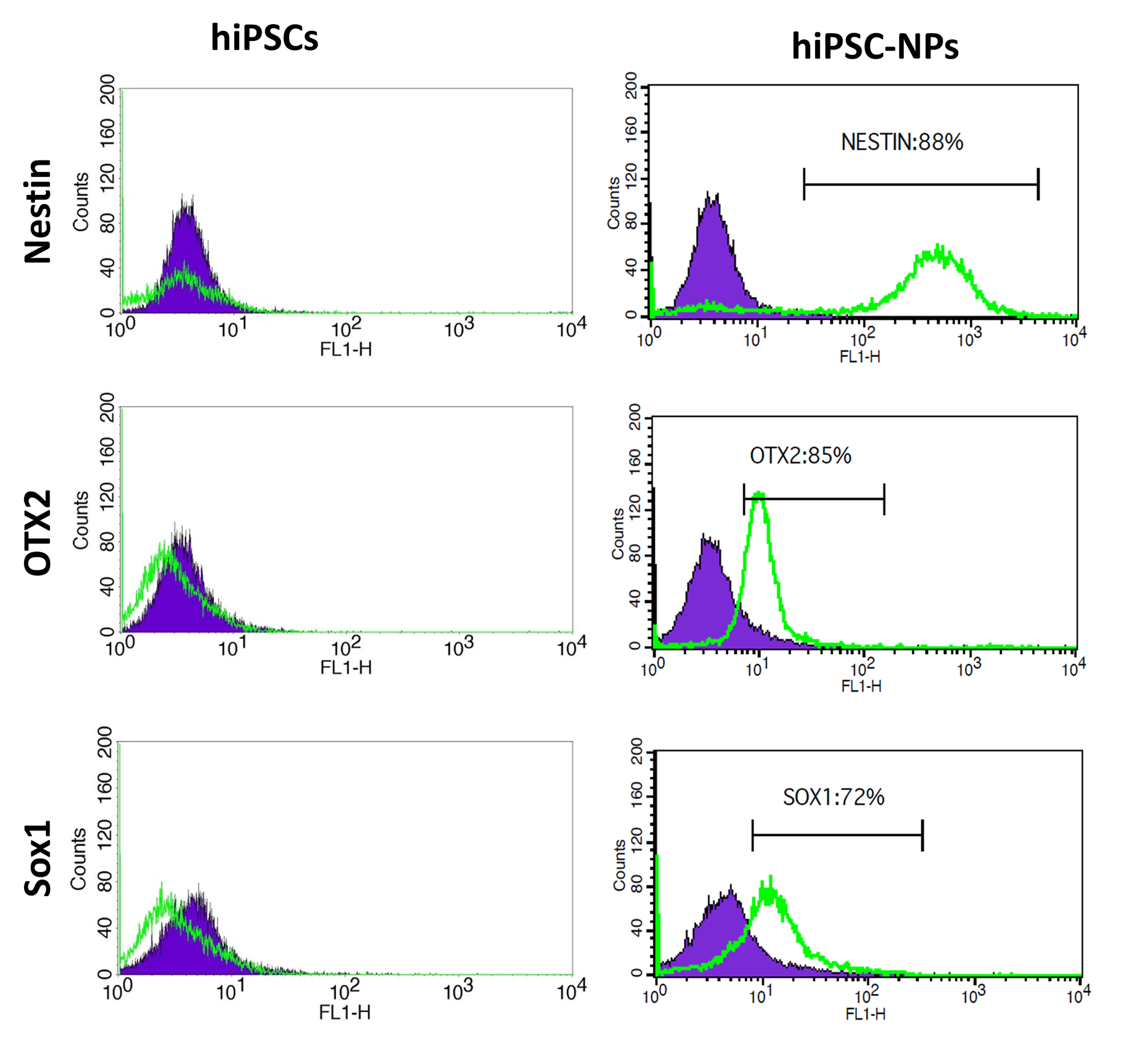

Supplement: Figure S1 — The histograms of hiPSc-NPs and hiPSCs analysis by flow cytometry. The averaged data are presented as figure 2B. (TIF) [file pone.0071855.s001.tif]

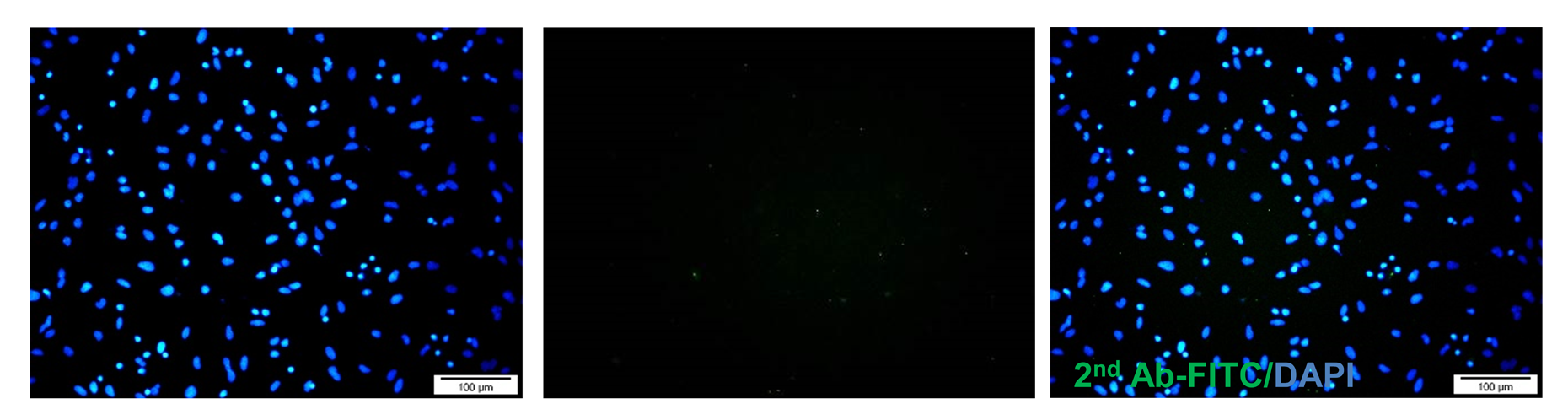

Supplement: Figure S2 — Immunofluorescence assessment to rule out non-specific staining by second antibody (2nd Ab-FITC). (TIF) [file pone.0071855.s002.tif]

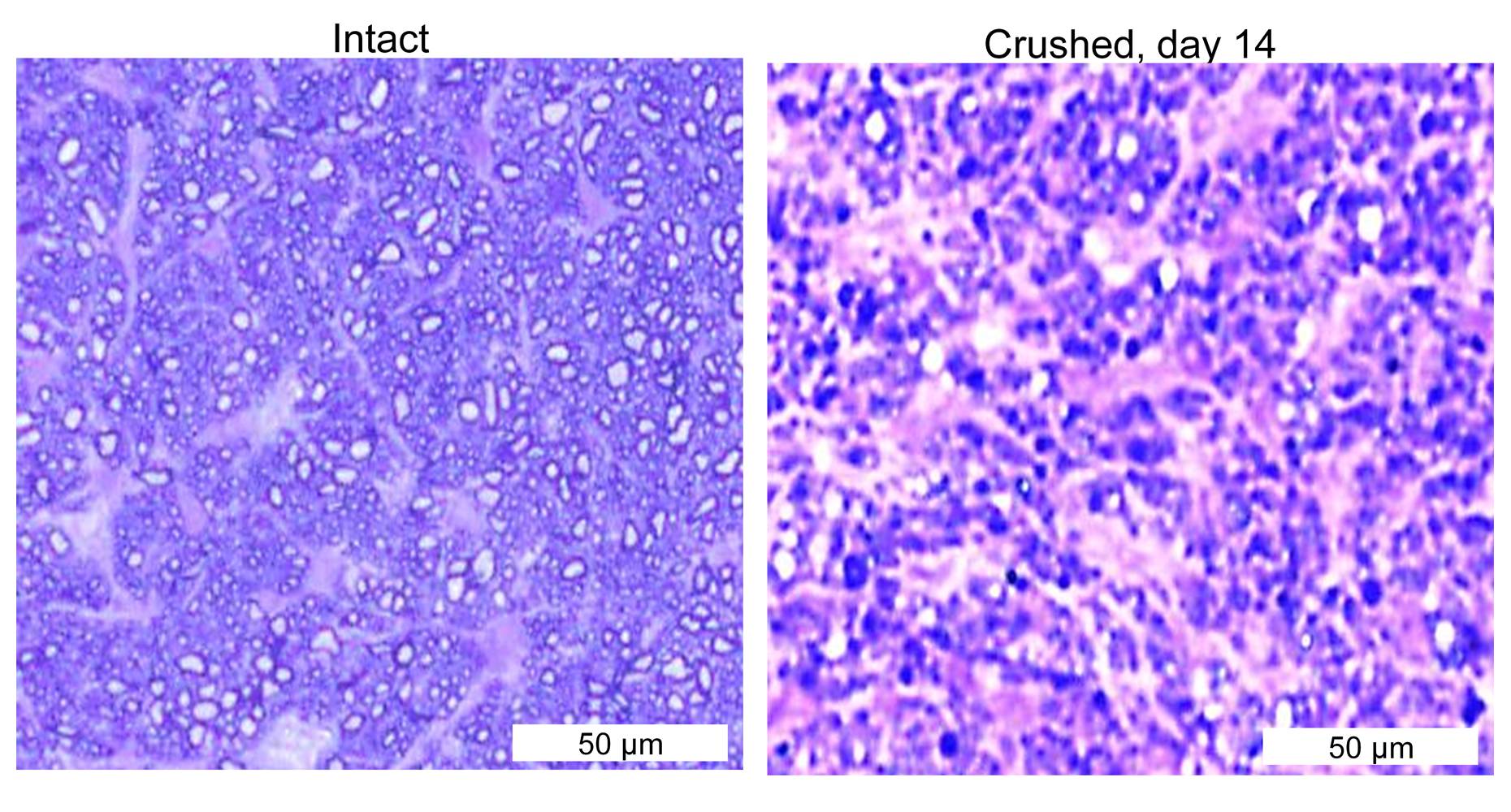

Supplement: Figure S3 — Semi-thin cross sections of optic nerves. For verifying the success of the optic nerve crush (ONC), we used toluidine blue staining of osmium tetroxide-embedded optic nerves that were obtained from intact or modeled animals on day 14 post-lesion. The staining protocol is described in the main text. Representative high magnification micrographs of healthy control and crushed optic nerve at two weeks post-lesion. Semi-thin cross sections of crushed optic nerves showed more than 75% axonal loss and extensive demyelination. (TIF) [file pone.0071855.s003.tif]

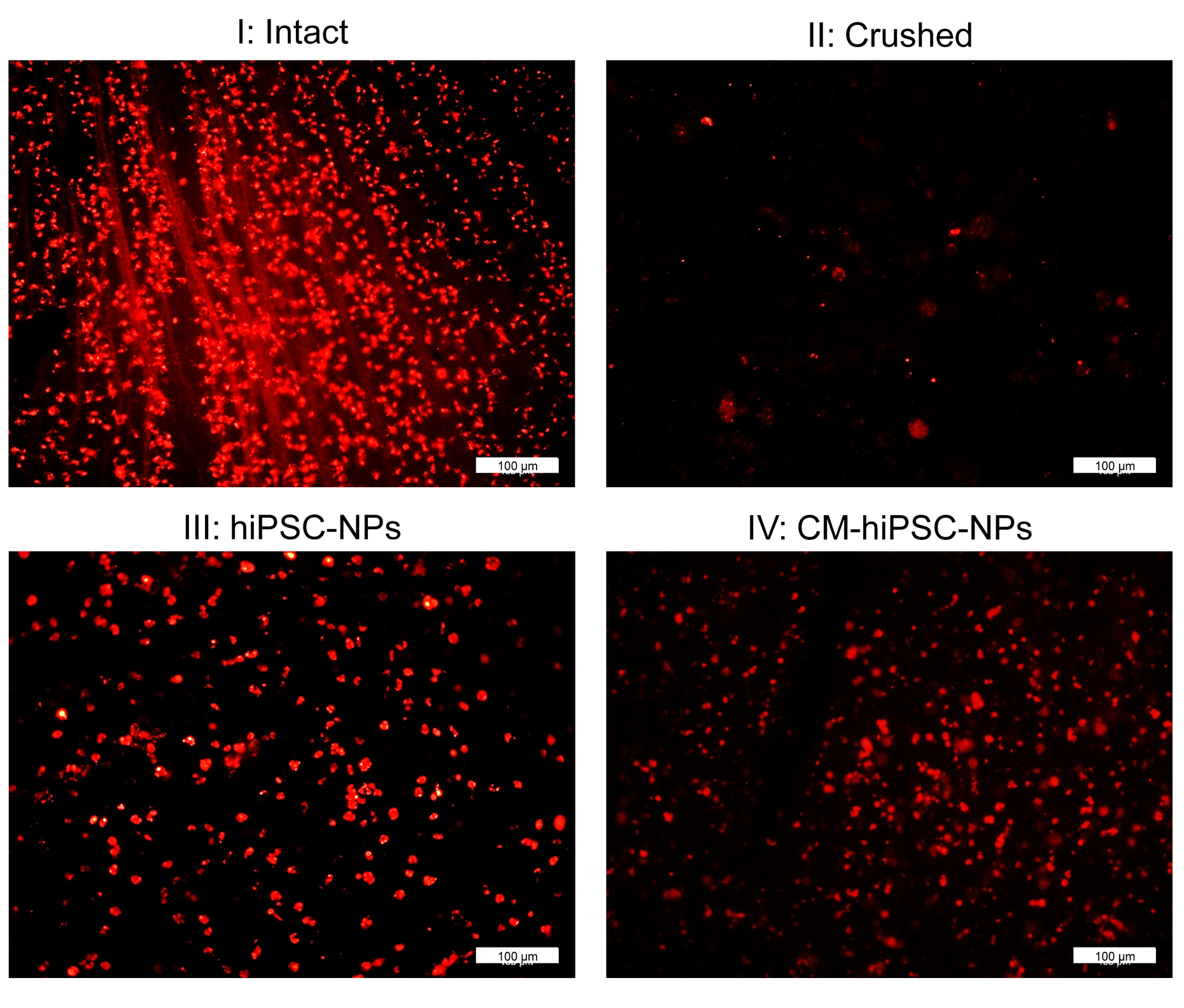

Supplement: Figure S4 — Retrograde-labeled RGCs. RGCs were retrogradely labeled using DiI injection into the superior colliculi for comparing the number of RGCs with intact axons in different groups. Treatment with both hiPSc-NPs and conditioned medium (CM) were able to increase the protected cells. Low power figures and quantitative data are mentioned in Figure 4 A and B. (TIF) [file pone.0071855.s004.tif]

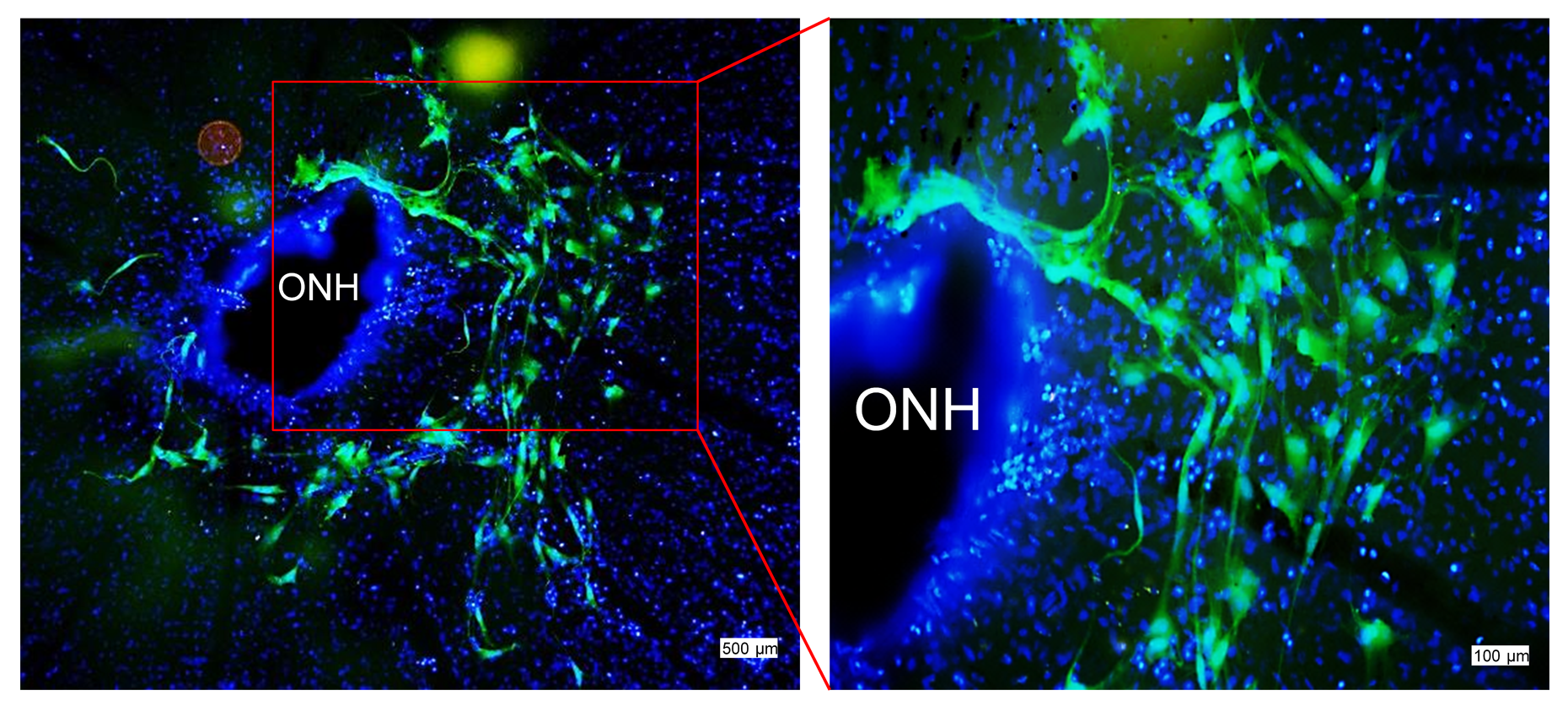

Supplement: Figure S5 — The integration of hiPSC-NPs into the retina of rat eyes with optic nerve crush. GFP-labeled hiPSC-NPs were traced at day 30 post-transplantation. Whole mount retina preparation of transplanted eye shows the integration of labeled cells into the retina. Transplanted cells showed neural morphology with considerable neurite outgrowths. Data for neural differentiation of the GFP-labeled transplanted cells are presented in Figure 6. (TIF) [file pone.0071855.s005.tif]

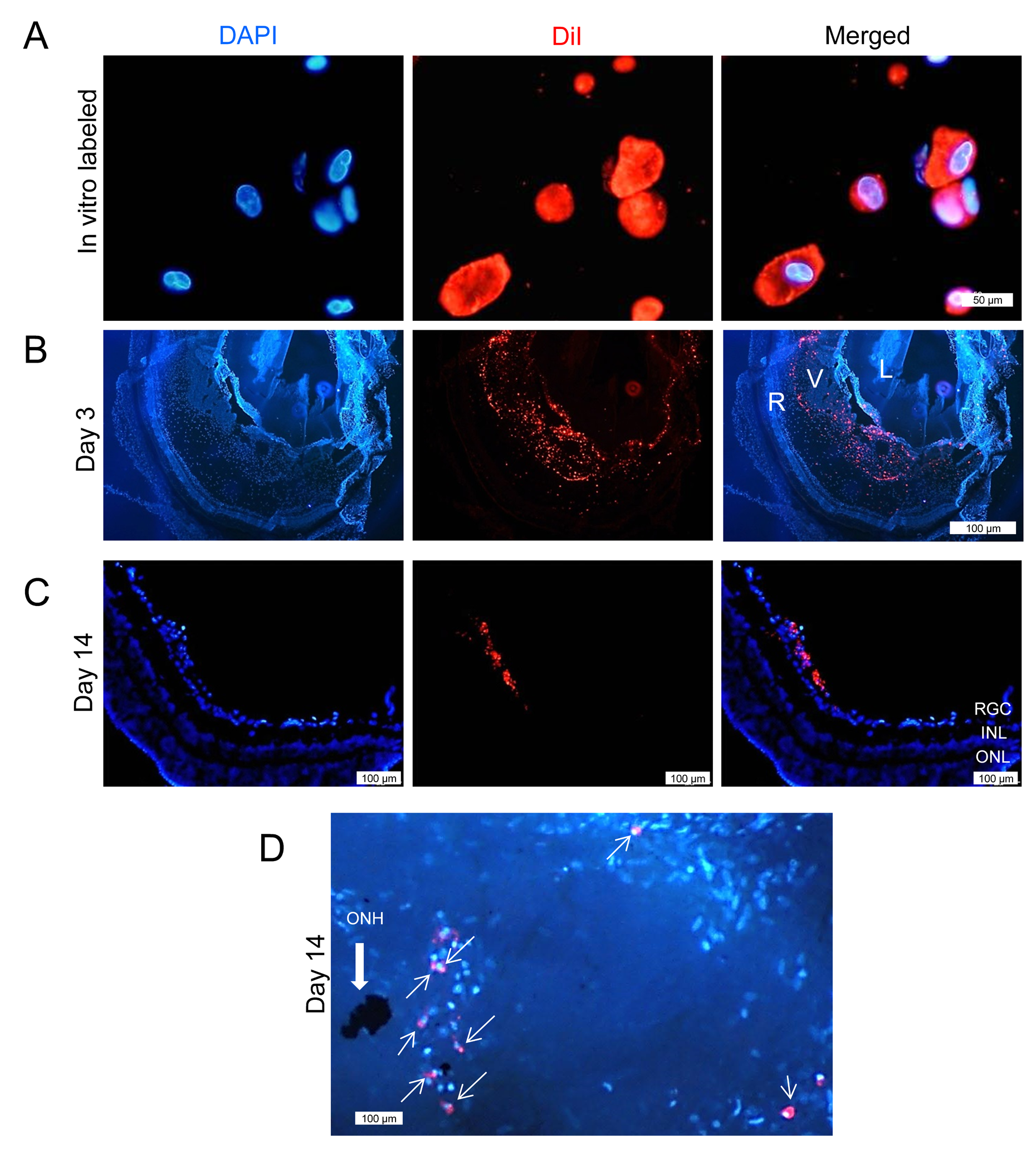

Supplement: Figure S6 — Fluorescent labeling of hiPSC-NPs and their localization after transplantation into the retina. (A) The labeled cells in vitro. (B) Large clusters of DiI-labeled hiPSC-NPs that survived within the vitreous after three days. (C) Some transplanted hiPSC-NPs migrated and localized in the proximity of the RGC layer at day 14 post-transplantation. (D) Whole mounted retina visualized 14 days after cell transplantation shows integrated cells. Arrows show transplanted cells and blue shows the nuclear staining using DAPI. L; Lens, V; Vitreous, R; Retina, ONL; Outer nuclear layer, INL; Inner nuclear layer, RGC; Retinal ganglion cell layer; ONH, Optic nerve head. (TIF) [file pone.0071855.s006.tif]

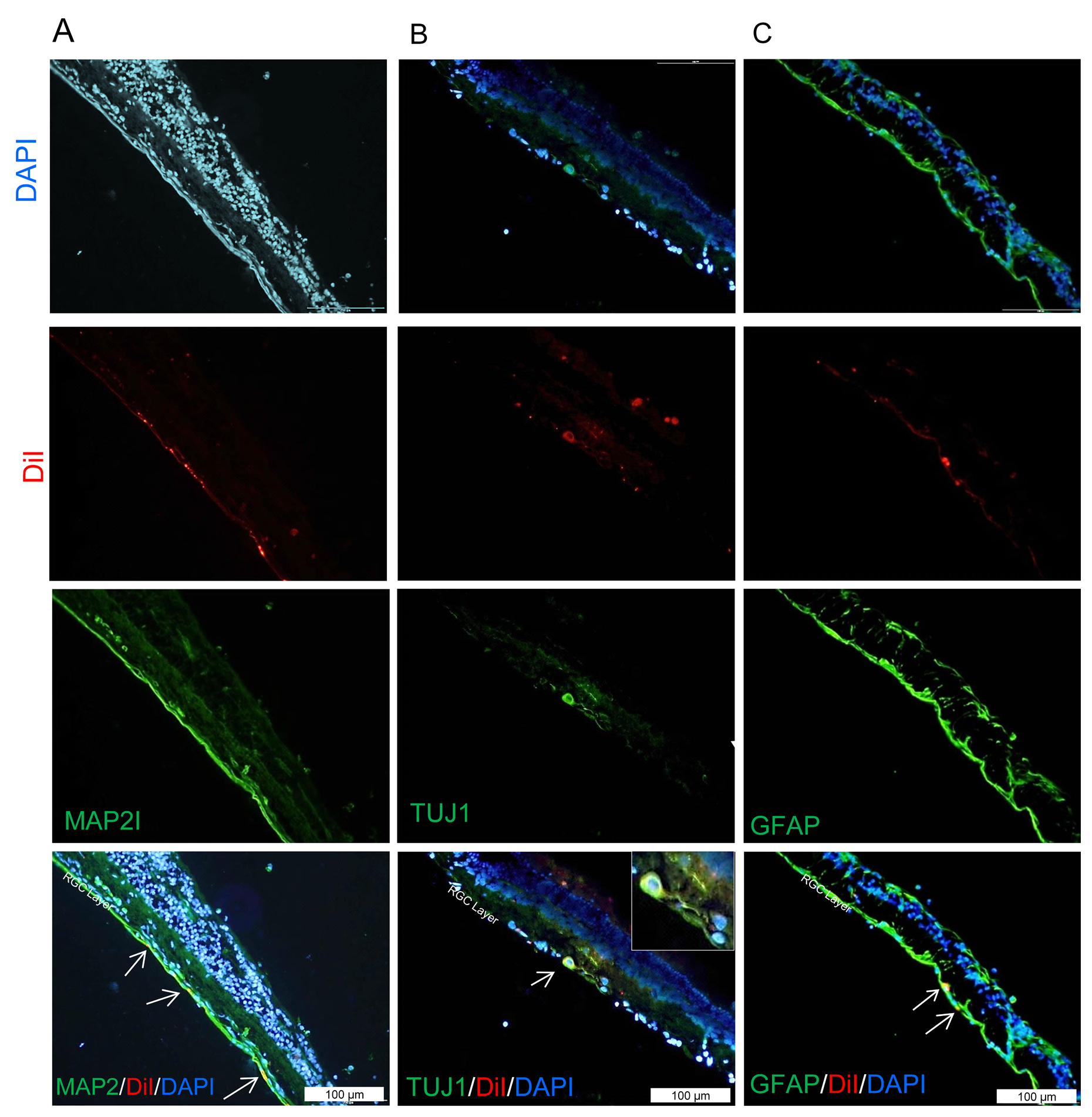

Supplement: Figure S7 — The integration and differentiation of hiPSC-NPs at 60 days after transplantation. Engrafted hiPSC-NPs were labeled by DiI (red fluorescent) and detected using immunohistoflourescence studies against neural markers MAPII and Tuj1 and counterstained with DAPI (blue). DiI+/MAPII+ cells or DiI+/Tuj+ (arrows) show that transplanted cells integrated into the RGC layer and underwent neural differentiation (A and B). Immunohistoflourescence evaluation of the retina sections confirmed that transplanted cells localized in the RGC layer and differentiated toward neurons and protruded fine neurite-like processes that elongated directly toward the optic nerve head (B, magnified in inlet). (C) Arrows show that some of the transplanted cells could participate in inner limiting membrane (ILM) repair and expressed the astrocyte/Muller cell marker GFAP. ONL; Outer nuclear layer, INL; Inner nuclear layer, RGC; Retinal ganglion cell layer. (TIF) [file pone.0071855.s007.tif]
