# Supplementary material for: Engrafted Human Induced Pluripotent Stem Cell-Derived Anterior Specified Neural Progenitors Protect the Rat Crushed Optic Nerve
Source: PLoS One. 2013 Aug 19;8(8):e71855. doi: 10.1371/journal.pone.0071855 (PMC3747054; doi:10.1371/journal.pone.0071855)
Supplement: Table S1 — Details of primers used for real time-PCR. (PDF) [file pone.0071855.s008.pdf]

Supplementary information

Table S1. Details of primers used for real time-PCR.

Gene	Primer sequences (5′-3′)	Accession no.
NESTIN	F: CTCCAGAAACTCAAGCACC	NM_006617
	R: TCCTGATTCTCCTCTTCCA	
SOX1	F: CCTCCGTCCATCCTCTG	NM_ 005986
	R: AAAGCATCAAACAACCTCAAG	
PAX 6	F: CGGTTTCCTCCTTCACAT	NM_000280.3
	R: ATCATAACTCCGCCCAT	
GAPDH	F: TCATTTCCTGGTATGACAACGA	N M_002046.3
	R: CTTCCTCTTGTGCTCTTGCT	
