# Supplementary material for: Engrafted Human Induced Pluripotent Stem Cell-Derived Anterior Specified Neural Progenitors Protect the Rat Crushed Optic Nerve
Source: PLoS One. 2013 Aug 19;8(8):e71855. doi: 10.1371/journal.pone.0071855 (PMC3747054; doi:10.1371/journal.pone.0071855)
Supplement: Table S2 — Details of antibodies and fluorescent markers. (PDF) [file pone.0071855.s009.pdf]

Table S2. Details of antibodies and fluorescent markers.

Specificity	Source	Type/label	Dilution	Company, Cat. NO
NESTIN	Mouse	Monoclonal	1: 200	Chemicon, MAB5326
SOX 1	Rabbit	Monoclonal	1: 50	Sigma-Aldrich, S8318
OTX 2	Rabbit	Monoclonal	1: 100	Sigma-Aldrich, HPA000633
PAX 6 (H-295)	Rabbit	Polyclonal	1: 200	Santa Cruz, 11357
MAPII	Mouse	Monoclonal	1: 200	Sigma Aldrich, M1406
B-TubulinIII	Mouse	Monoclonal	1: 250	Sigma Aldrich, T8660
GFAP	Mouse	Monoclonal	1: 400	Sigma Aldrich, G3893
S100	Rabbit	Monoclonal	1: 300	Santa Cruz, 2644
GAP43	Mouse	Monoclonal	1: 500	Invitrogen, 33-5000
NF200	Mouse	Monoclonal	1: 200	Sigma Aldrich, 0142
Fluoromyelin	-	-	1: 300	Molecular probes,F34652
Anti-Mouse IgG	Goat	FITC	1: 100	Chemicon, AP308F
Anti-Rabbit IgG	Goat	FITC	1: 100	Sigma Aldrich, F1262
Anti-Mouse IgG	Goat	FITC	1: 100	Sigma Aldrich, F9006
Anti-Rabbit IgG	Goat	Alexa fluor	1: 1000	Molecular probe, 11037
Anti-Mouse IgG	Goat	Alexa fluor	1: 1000	Molecular probe, 11005
