# Supplementary material for: Engrafted Human Induced Pluripotent Stem Cell-Derived Anterior Specified Neural Progenitors Protect the Rat Crushed Optic Nerve
Source: PLoS One. 2013 Aug 19;8(8):e71855. doi: 10.1371/journal.pone.0071855 (PMC3747054; doi:10.1371/journal.pone.0071855)
Supplement: Results S1 — Determining the fate of grafted cells in the host retina using DiI labeling. (PDF) [file pone.0071855.s010.pdf]

Results S1.

Determining the fate of grafted cells in the host retina using DiI labeling

Prepared hiPSC-NPs for transplantation were labeled with a red fluorescence-cell tracer, CM-DiI (chloromethyl benzamido derivatives of 1, 1-dioctadecyl-3, 3, 3´, 3´ tetramethylindocarbocyanine perchlorate; Invitrogen, C7001) prior to transplantation. Cells were incubated in expansion medium that contained 5 µg/ml of CM-DiI for 20 min in a cell culture incubator. Then, cells were washed three times for 5 minutes each by D-PBS (Gibco-Invitrogen, 14190-144) and concentrated to 25 000 cells/µl.

In vitro fluorescent labeling using DiI was performed for later tracing of transplanted cells. Supplementary Figure 6A shows the labeled cells prior to transplantation. At three days post-transplantation the majority of grafted cells remained as a bolus within the vitreous cavity; some were in close vicinity to the RGCs layer (Supplementary Fig. 6B). Supplementary Figure 6C shows the sections of transplanted eyes on day 14 post-transplantation. While the majority of cells were removed from the vitreous space, some transplanted cells were attached to the retina as clusters. Compared to the third day, the numbers of transplanted cells were dramatically decreased. Migration of NPs into the retina was also examined on flat whole mount retina at day 14th post-transplantation. Supplementary Figure 6D shows some of the transplanted cells inside the whole mount preparation of the retina.

To determine whether these NPs could acquire a neural fate in vivo and integrate into the retina, immunofluorescent staining against specific neural markers was performed at day 60 post-transplantation. Analysis of the sections revealed that the donor cells expressed neural and glial markers. At this time point, most of the injected cells which were detectable by DiI fluorescent dye had integrated within the RGCs layer. Some of these cells were immunoreactive for neuronal markers, MAPII (Supplementary Fig. 7A) and β-III tubulin (Supplementary Fig. 7B). Most of the integrated cells possessed a neuronal-like morphology and showed the extension of numerous fine processes which projected horizontally in the direction of the optic nerve head. A small portion of integrated cells seemed to express GFAP as an astrocyte marker (Supplementary Fig. 7C).
